# CHOP Mediates Endoplasmic Reticulum Stress-Induced Apoptosis in Gimap5-Deficient T Cells

**DOI:** 10.1371/journal.pone.0005468

**Published:** 2009-05-08

**Authors:** Steven C. Pino, Bryan O'Sullivan-Murphy, Erich A. Lidstone, Chaoxing Yang, Kathryn L. Lipson, Agata Jurczyk, Philip diIorio, Michael A. Brehm, John P. Mordes, Dale L. Greiner, Aldo A. Rossini, Rita Bortell

**Affiliations:** 1 Department of Medicine, University of Massachusetts Medical School, Worcester, Massachusetts, United States of America; 2 Molecular Medicine, University of Massachusetts Medical School, Worcester, Massachusetts, United States of America; LMU University of Munich, Germany

## Abstract

Gimap5 (GTPase of the immunity-associated protein 5) has been linked to the regulation of T cell survival, and polymorphisms in the human *GIMAP5* gene associate with autoimmune disorders. The BioBreeding diabetes-prone (BBDP) rat has a mutation in the *Gimap5* gene that leads to spontaneous apoptosis of peripheral T cells by an unknown mechanism. Because Gimap5 localizes to the endoplasmic reticulum (ER), we hypothesized that absence of functional Gimap5 protein initiates T cell death through disruptions in ER homeostasis. We observed increases in ER stress-associated chaperones in T cells but not thymocytes or B cells from *Gimap5^−/−^* BBDP rats. We then discovered that ER stress-induced apoptotic signaling through C/EBP-homologous protein (CHOP) occurs in *Gimap5^−/−^* T cells. Knockdown of CHOP by siRNA protected *Gimap5^−/−^* T cells from ER stress-induced apoptosis, thereby identifying a role for this cellular pathway in the T cell lymphopenia of the BBDP rat. These findings indicate a direct relationship between Gimap5 and the maintenance of ER homeostasis in the survival of T cells.

## Introduction

The expression products of the GTPase of the immunity-associated gene family (gimaps; formerly known as IANs or immune-associated nucleotide-binding proteins) have been implicated in the regulation of T cell survival through modulation of T cell receptor (TCR) signaling [1], [2]. A member of this family, *GIMAP5*, has an important role in human immune modulation as a polyadenylation polymorphism in the human *GIMAP5* gene increases systemic lupus erythematosus risk [3] and is also associated with elevated islet autoantibodies in type 1 diabetics [4]. Gimap protein may also play a significant role in T cell homeostasis because peripheral T cell survival is impaired in both *gimap5* null mice [5] and in BioBreeding diabetes-prone (BBDP) rats, which have a frameshift mutation in the *Gimap5* gene [6]–[8]. In the BBDP rat, Gimap5 is a truncated protein in which the carboxy-terminal 215 amino acids are replaced by 19 other amino acids. Absence of functional wild type Gimap5 protein results in mitochondrial dysfunction and apoptosis *in vitro*, and severe T cell lymphopenia *in vivo* [9]–[11]. Knockdown of human Gimap5 in Jurkat T cells *in vitro* recapitulates the natural apoptosis that occurs in T cells from the BBDP rat [11]. However, the mechanism by which *Gimap5* promotes T cell survival is not understood.

Gimap5 has been reported to localize mainly to the endoplasmic reticulum (ER) [12], [13] but is also detected in mitochondria, the Golgi apparatus, and centrosomes [13], [14]. The ER is a multifunctional organelle which plays a vital role in the regulation of numerous cellular processes, including synthesis, folding, and assembly of secretory and transmembrane proteins [15], [16], calcium (Ca^2+^) signaling regulation, vesicle trafficking, drug metabolism, and lipid biogenesis [17]–[19]. As the main organelle involved in protein processing within a cell, the ER has evolved numerous signaling pathways that monitor its protein folding capacity and ensure that these pathways do not become dysfunctional [16]. Pathological disturbances to normal cellular functioning, including disruption of protein folding or alterations in Ca^2+^ homeostasis [20], interfere with the functioning of the ER and cause stress to the organelle.

Normally, stress within the ER triggers an adaptive cellular mechanism known as the unfolded protein response or ER stress response that attempts to return the cell to homeostasis [21], [22]. Activation of the ER stress response is accomplished by specific ER transmembrane proteins that function as ER stress transducers [23]. Glucose-regulated protein 78 (GRP78) is the central regulator of the ER stress response. It normally binds to and inhibits signaling from ER stress transducers. However, during ER stress GRP78 is released from those transducers, thereby activating the ER stress response [24]. If ER stress persists and cellular homeostasis cannot be restored, the ER stress response can initiate cell death stimuli that lead to ER stress-induced apoptosis [18].

Initiation of ER stress-induced apoptosis through ER stress response signaling involves transcriptional activation of the bZIP transcription factor *chop* (C/EBP-homologous protein) [25]. CHOP acts to repress the promoter of the *bcl-2* gene, thus downregulating antiapoptotic Bcl-2 protein and rendering cells sensitive to the proapoptotic effects of BH3-only proteins [26]. Because Gimap5 localizes primarily to the ER [12], [13], we hypothesized that Gimap5 protein in T cells is necessary to maintain ER homeostasis. In this report, we show that ER stress response signaling occurs in *Gimap5^−/−^* T cells as evidenced by an increase in ER chaperone expression. We further show that initiation of ER stress-induced apoptotic signaling in *Gimap5^−/−^* T cells is associated with an increase of CHOP protein. Knockdown of CHOP expression protects *Gimap5^−/−^* T cells from ER stress-induced cell death, thus revealing an essential role for Gimap5 in the maintenance of ER homeostasis and T cell survival.

## Results

### Increased expression of ER stress response proteins in Gimap5^−/−^ BBDP rat lymphocytes

**Figure 1:**
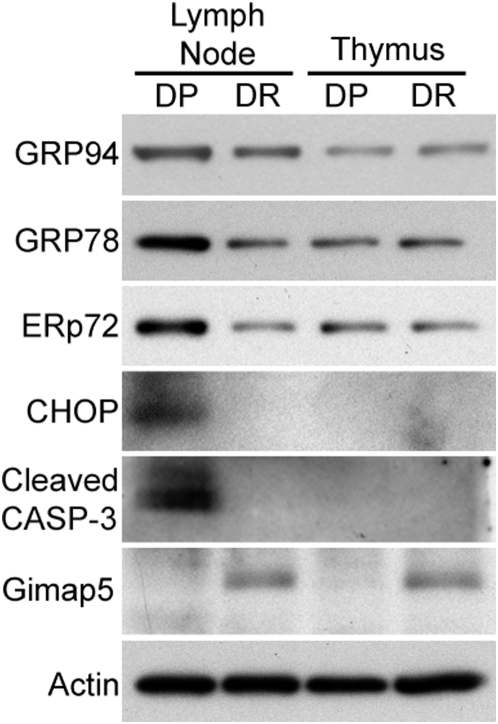
The expression of ER chaperones increases in lymphocytes from the Gimap5^−/−^ BBDP rat. Western blot analyses of ER chaperone proteins GRP94, GRP78, and ERp72; the ER stress apoptotic factor CHOP; and cleaved caspase-3 (CASP-3) in lymphocytes and thymocytes from BBDP and BBDR rats. Gimap5 protein expression was confirmed in lysates from BBDR rats and actin was used as a loading control. Data shown are representative of three independent experiments.

Gimap5 is normally expressed in the thymus and in both T and B cells of lymphoid tissues [11]. However, depletion of functional Gimap5, whether by RNAi, genetic knockout or mutation, leads to spontaneous apoptosis only in T cells [5], [11]. To examine the potential role of ER stress in T cell death of *Gimap5^−/−^* rats, we compared the expression of ER chaperone proteins in lymph node cells and thymocyte lysates from *Gimap5^−/−^* BBDP and *Gimap5^+/+^* BioBreeding diabetes-resistant (BBDR) rats. Western blot analyses revealed that GRP94, GRP78, and ER protein 72 (ERp72) levels were increased in lymphocytes from *Gimap5^−/−^* BBDP rats when compared to that in *Gimap5^+/+^* BBDR rats (Fig. 1). However, ER chaperone expression was similar in thymocytes from *Gimap5^−/−^* BBDP and *Gimap5^+/+^* BBDR rats. These results indicate that the ER stress response leading to increased ER chaperone expression is specifically upregulated in lymph node cells and not thymocytes in *Gimap5^−/−^* BBDP rats.

To determine if ER stress-induced apoptosis was also activated in *Gimap5^−/−^* lymph node cells, we examined expression of the ER stress apoptotic factor, CHOP. CHOP expression is minimal and difficult to detect under normal homeostatic conditions [27], and upregulation of CHOP has been reported to signal the activation of ER stress-mediated apoptotic signals [25]. These signals ultimately culminate in cell death through proteolytic cleavage of caspase-3 [28]. We found that CHOP protein is abundantly expressed in lymph node cells from *Gimap5^−/−^* BBDP rats, but is not detectable in BBDP thymocytes or in thymocytes or lymphocytes from *Gimap5^+/+^* BBDR rats (Fig. 1). Furthermore, the presence of activated (cleaved) caspase-3, the executor of apoptosis downstream of ER stress-mediated apoptotic signaling, was only detected in *Gimap5^−/−^* BBDP lymphocytes. As expected, Gimap5 protein was not detected in lymph node cells from *Gimap5^−/−^* BBDP rats, but was expressed at comparable levels in both lymph node cells and thymocytes from *Gimap5^+/+^* BBDR rats (Fig. 1). These data provide evidence of an ER stress response and ER stress-induced apoptotic signaling that is specific to lymph node cells from *Gimap5^−/−^* BBDP rats.

### Increased GRP78 and CHOP in both CD4^+^ and CD8^+^ *Gimap5^−/−^* BBDP T cells

**Figure 2:**
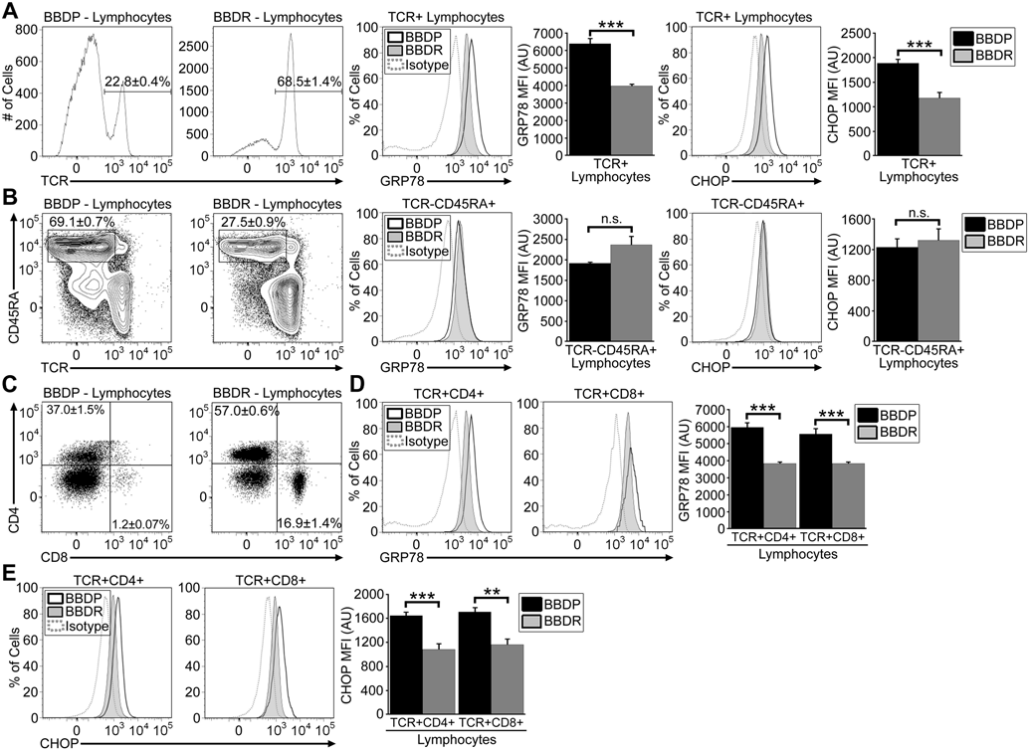
*Gimap5^−/−^* BBDP rat T cell populations exhibit enhanced ER stress response signaling. Representative analyses showing the gating of TCR^+^ (A), TCR^−^CD45RA^+^ (B), and CD4^+^ or CD8^+^ (C) lymphocytes from BBDP and BBDR rats. Above the representative gate is shown the average percentage for that cell type from 4 rats of each strain (±SEM). Representative histograms show the percentage of cells expressing intracellular GRP78 and CHOP protein at the indicated levels in BBDP (black line) and BBDR (shaded region) rat T cells (A), B cells (B), and CD4^+^ or CD8^+^ T cells (D,E). The corresponding isotype control is depicted as a dotted line in each histogram. Bar graphs display the MFI in arbitrary units (AU) of intracellular GRP78 and CHOP protein in each cell type (±SEM). Data shown are representative of three independent experiments (**P<.01; *** *P*<.001).

Because BBDP rats have a peripheral T cell lymphopenia that, in transgenic rescue experiments [29], has been proven to be due to its mutation in the *Gimap5* gene [9], [10], [30], we hypothesized that the increased levels of ER stress proteins found in BBDP rat lymph node cells preparations (Fig. 1) would be associated specifically with the T cell compartment. As expected, we found a decreased percentage of TCR^+^ cells in the lymph nodes of BBDP rats (22.8±0.4%) as compared to Gimap5^+/+^ BBDR rats (68.5±1.4%, n = 4; *P*<0.001, Fig. 2A). We also observed a concomitant enrichment of B cells (TCR^−^CD45RA^+^) in BBDP (69.1±0.7%) vs BBDR rats (27.5±0.9%, n = 4; *P*<0.001, Fig. 2B). In cervical and mesenteric lymph nodes, the absolute number of T cells in the BBDP rat (23.9±2.2×10^6^) was significantly less than in the *Gimap5^+/+^* BBDR rats (123.3±15.0×10^6^, n = 4; *P*<0.01), whereas B cell numbers were significantly increased (75.6±5.2×10^6^ vs 50.0±6.2×10^6^, n = 4; *P*<0.05). To determine the T cell-specific levels of ER stress response and ER stress-induced apoptotic signaling, we performed intracellular flow cytometry. As measured by the mean fluorescence intensity (MFI), both GRP78 and CHOP levels were higher in TCR^+^ lymphocytes from *Gimap5^−/−^* BBDP rats than from *Gimap5^+/^* ^+^ BBDR rats (Fig. 2A). In contrast, B lymphocytes (TCR^−^CD45RA^+^ cells) exhibited no difference in GRP78 and CHOP expression between the two rat strains (Fig. 2B).

We next examined ER stress response activation within peripheral CD4^+^ and CD8^+^ T cells. Consistent with previous observations [30], we found that the CD8^+^ T cell subset in BBDP rats (1.2±0.07% vs 16.9±1.4% in BBDR, n = 3) was reduced to a much greater degree than was the CD4^+^ subset (37.0±1.5% vs 57.0±0.6% in BBDR, n = 4) (Fig. 2C). Absolute CD4^+^ T cell number in lymph node cells were 40.4±4.0×10^6^ cells in the BBDP rat vs 102.5±11.7×10^6^ in the BBDR (n = 4; *P*<0.01). Absolute CD8^+^ T cell number in lymph node cells were 1.4±0.1×10^6^ in the BBDP rat vs 30.6±4.5×10^6^ in the BBDR, (n = 4; *P*<0.01).

To determine whether ER stress levels are higher in the CD8^+^ subset, we again analyzed GRP78 and CHOP by intracellular flow cytometry. We observed that GRP78 and CHOP expression was similar in both CD4^+^ and CD8^+^ T cells from the *Gimap5^−/−^* BBDP rat, but both subsets of BBDP T cells had GRP78 and CHOP levels that were higher than their counterparts from the *Gimap5^+/+^* BBDR rat (Fig. 2D,E). These data suggest that the ER stress response and ER stress-induced apoptotic signaling are activated in both CD4^+^ and CD8^+^ T cells in the BBDP rat.

### Increased ER stress response signaling occurs when *Gimap5^−/−^* T cells first exit the thymus

**Figure 3:**
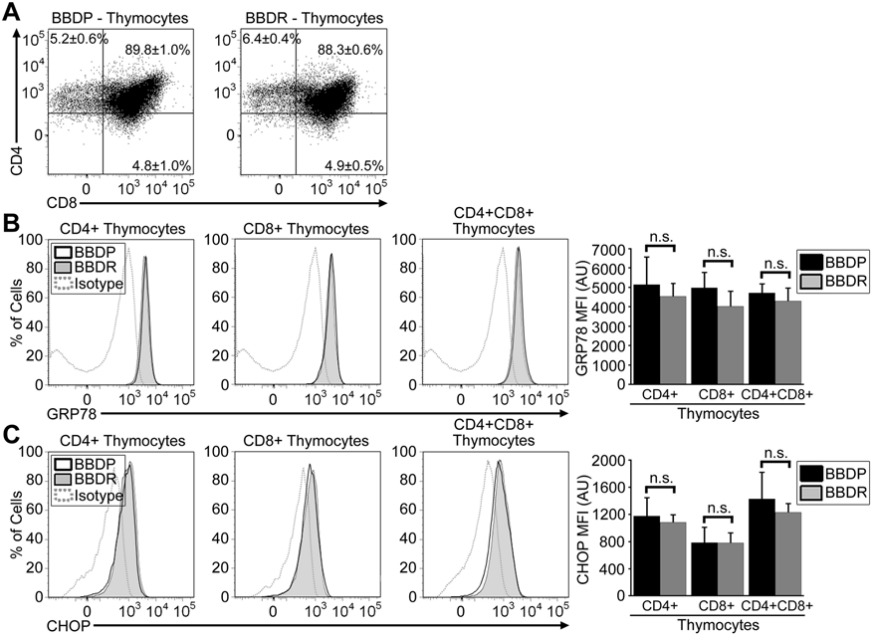
ER stress response signaling is similar in thymocytes from *Gimap5^−/−^* BBDP and *Gimap5^+/+^* BBDR rats. (A) Representative flow cytometric dot plots depicting CD8 expression (horizontal axis) and CD4 expression (vertical axis) on thymocytes. Numbers represent the percentage of cells (±SEM of triplicate samples) in each gate shown. Representative histograms depicting the expression of intracellular GRP78 (B) and CHOP (C) protein in thymocyte populations from BBDP (black line) and BBDR (shaded region) rats. Isotype control for GRP78 or CHOP staining (dotted line) is shown in each histogram. Bar graphs display the MFI in arbitrary units (AU) of intracellular GRP78 or CHOP protein expression (±SEM). Data shown are representative of three independent experiments.

Thymocyte development in the Gimap5^−/−^ BBDP rat is generally normal [31], with typical differentiation of double positive CD4^+^CD8^+^ cells to single positive thymocyte precursors [30]. Consistent with previous reports [30], our flow cytometry analyses revealed similar percentages of CD4^+^CD8^+^, CD4^+^CD8^−^, and CD4^−^CD8^+^ thymocytes in both BBDP and BBDR rats (BBDP: 89.8±1.0%, 5.2±0.6%, 4.8±1.0%; BBDR: 88.3±0.6%, 6.4±0.4%, 4.9±0.5%, respectively, n = 3; Fig. 3A). Western blot analyses (Fig. 1) revealed no substantial difference in ER stress proteins in thymocytes of BBDP vs BBDR rats. To determine if enhanced ER stress signaling might be initiated in only a small subpopulation of thymocytes, we employed flow cytometry. Comparison of thymocytes isolated from BBDP and BBDR rats revealed no significant difference in GRP78 and CHOP levels within the CD4^+^CD8^+^, CD4^+^CD8^−^, and CD4^−^CD8^+^ thymocyte subsets (Fig. 3B,C). These data suggest that enhanced ER stress response signaling is *not* initiated in *Gimap5^−/−^* thymocytes prior to thymic export.

**Figure 4:**
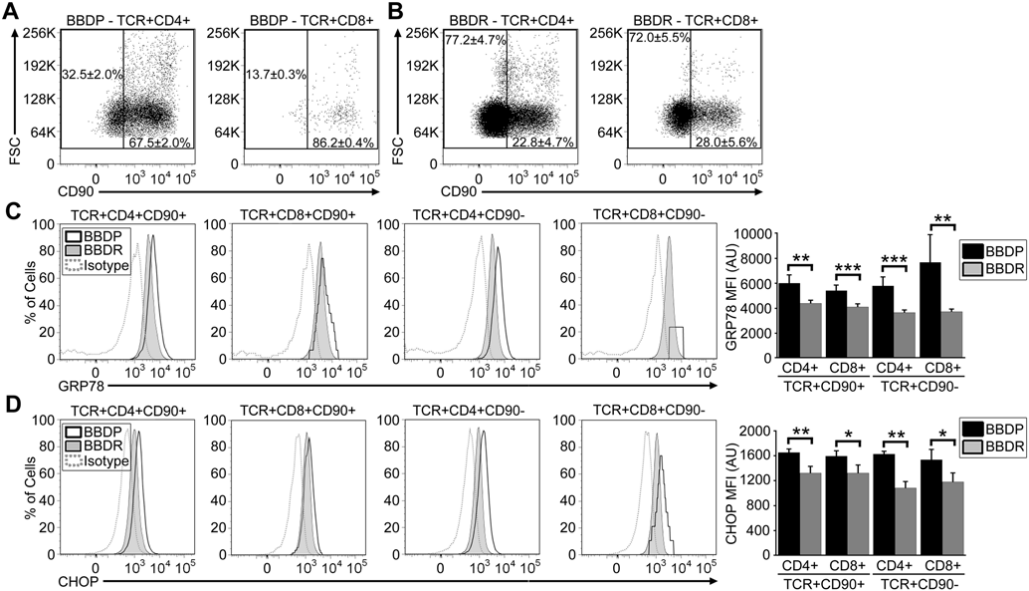
Enhanced ER stress response signaling is initiated in RTEs of the *Gimap5^−/−^* BBDP rat and continues during maturation to the CD90^−^ stage. (A,B) Representative flow cytometric dot plots depicting CD90 expression (horizontal axis) and forward scattering (vertical axis) of lymphocytes within gated CD4^+^ and CD8^+^ T cell populations in BBDP or BBDR rats. Numbers represent the corresponding average of CD90^−^ and CD90^+^ T cells (±SEM of triplicate samples). The expression of intracellular GRP78 (C) or CHOP (D) protein in representative histograms (BBDP, black line; BBDR, shaded region; isotype control staining, dotted line). The MFI of GRP78 or CHOP protein expression is indicated in bar graphs with error bars representing the SEM. Data shown are representative of three independent experiments (*P<.05; **P<.01; *** *P*<.001).

To determine the precise stage in CD4^+^ and CD8^+^ T cell development at which enhanced ER stress signaling is initiated, we next compared GRP78 and CHOP levels in mature T cells and recent thymic emigrants (RTEs, identified by the expression of CD90/Thy-1). Flow cytometry demonstrated that the preponderance of CD4^+^ and CD8^+^ T cells from *Gimap5^−/−^* BBDP rats still express CD90 on the cell surface, indicating that few T cells survive to the mature CD90^−^ stage (Fig. 4A). In contrast, the majority of CD4^+^ and CD8^+^ T cells from the *Gimap5^+/+^* BBDR rats mature and down-regulate CD90 (Fig. 4B). CD4^+^ and CD8^+^ RTEs from BBDP rats initiate significantly enhanced ER stress signals as compared to BBDR rats, as evidenced by increased GRP78 and CHOP expression (Fig. 4C,D). These increased levels of GRP78 and CHOP continue throughout T cell development into the small population of mature CD4^+^ and CD8^+^ CD90^−^ T cells that survive in the *Gimap5^−/−^* BBDP rat (Fig. 4C,D). Similar findings were also observed in congenic WF.*ART2a.Gimap5^−/−^* rats that harbor the same recessive mutation in the *Gimap5* gene as do BBDP rats, but do not develop spontaneous diabetes (Figure S1). All T cell populations, except for TCR^+^CD8^+^CD90^+^ cells from WF.*ART2a.Gimap5^−/−^* rats, contained increased expression of GRP78 and CHOP, as compared to control WF.*ART2a.Gimap^+/+^* congenic rats. We attribute the difference in ER stress response signaling in the TCR^+^CD8^+^CD90^+^ population to genetic background differences which occur between the BB and WF rat strains. These results indicate that ER stress response signaling is not a secondary consequence of diabetes development. Overall, our data reveal a central role for Gimap5 protein in ER homeostasis during post-thymic development when T cells first enter the periphery.

### Enhanced GRP78 and CHOP expression is not dependent on T cell activation

**Figure 5:**
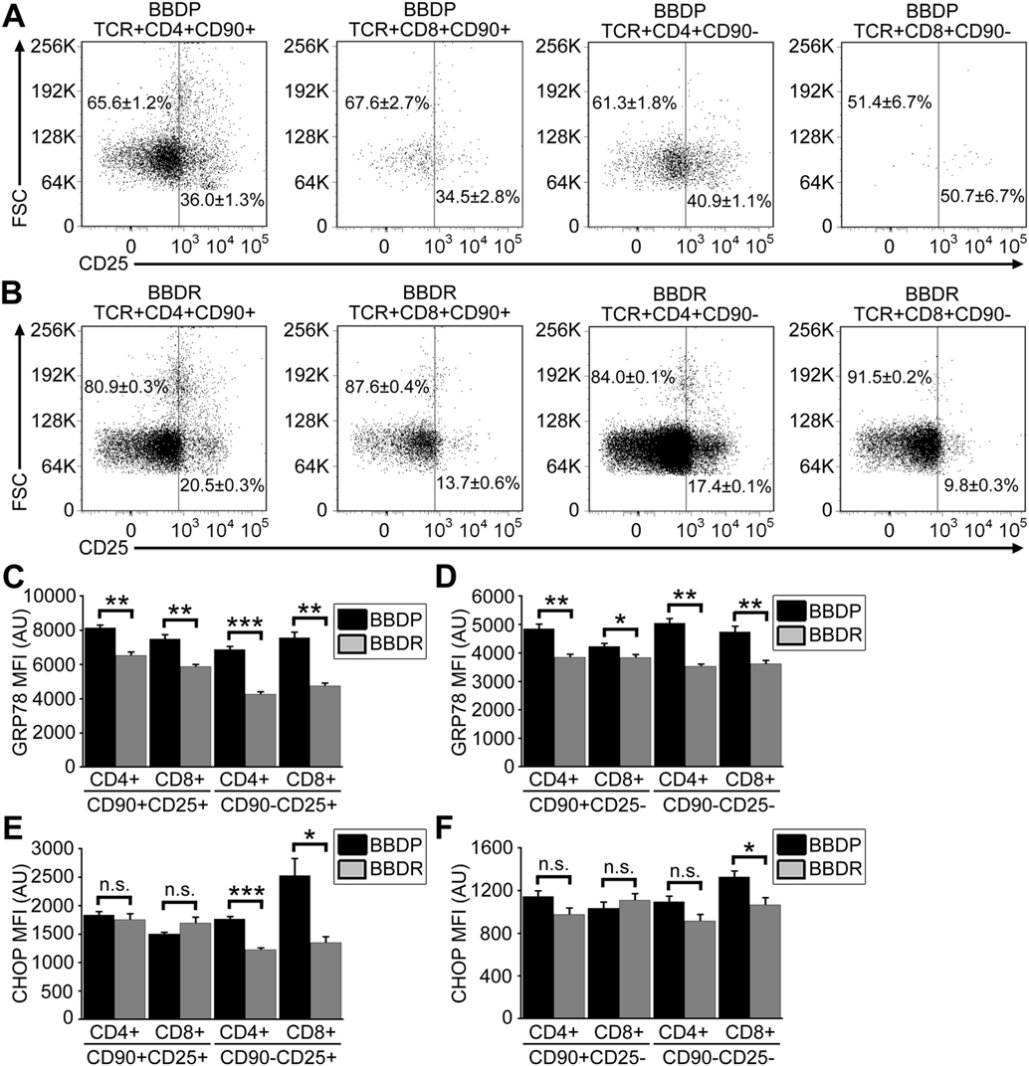
Enhanced ER stress response signaling in *Gimap5^−/−^* BBDP rat T cells is not dependent upon activation status. Representative flow dot plots depicting CD25 expression (horizontal axis) and forward scatter (vertical axis) of lymphocytes within gated CD4^+^ and CD8^+^ RTEs and mature T cells from BBDP (A) and BBDR (B) rats. Numbers represent the percentage (±SEM of triplicate samples) of corresponding CD25^−^ and CD25^+^ T cells. Bar graphs displaying the MFI of intracellular GRP78 and CHOP expression in CD25^+^ (C,E) and CD25^−^ (D,F) T cells. Error bars represent the SEM; data shown are representative of three independent experiments (*P<.05; **P<.01; *** *P*<.001).

BBDP rat T cells have been reported to exhibit an unusual state of partial activation following thymic export [2], [31]. Our data reveal a significant increase in the percentages of CD4^+^ (n = 3; *P*<0.001) and CD8^+^ (n = 3; *P* = 0.01) RTEs and mature CD4^+^ (n = 3; *P*<0.001) and CD8^+^ (n = 3; *P*<0.01) T cells from the *Gimap5^−/−^* BBDP rat that express CD25, a marker of activation (see Fig. 5A,B for representative data). Because we and others have shown that activation of T cells causes ER stress response signaling [32], [33], we wanted to determine if there was a link between cellular activation status and elevated GRP78 and CHOP expression in BBDP T cells. In the BBDP rat, CD4^+^ and CD8^+^ RTEs and mature T cells (CD90^−^) that express CD25 have significantly increased GRP78 expression as compared to cells with the same phenotype in the BBDR rat (Fig. 5C). However, the CD25^−^ T cells that survive in the BBDP rat also have significantly increased GRP78 levels as compared with CD25^−^ T cells from the BBDR rat (Fig. 5D). Expression of CHOP within CD25^+^ and CD25^−^ T cell populations was similar to that of GRP78 with respect to activation with one exception. Only CD4^+^CD90^−^CD25^+^ and CD8^+^CD90^−^CD25^+/−^ T cells from BBDP rats expressed significantly higher CHOP levels than did cells with the same phonotype from BBDR rats (Fig. 5E,F). These data indicate that, although ER stress levels do increase with activation status as previously reported, absence of functional Gimap5 protein is by itself sufficient to initiate ER stress response and ER stress-induced apoptotic signaling without concomitant T cell activation.

### Knockdown of CHOP protein expression in *Gimap5^−/−^* rat T cells inhibits apoptosis and cell death

**Figure 6:**
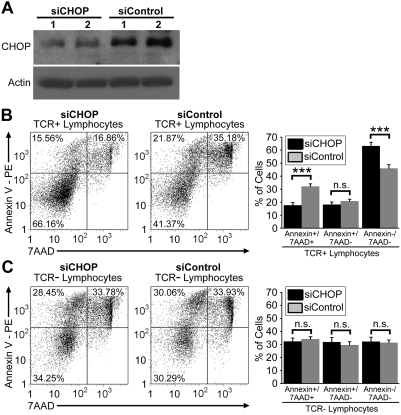
Knockdown of CHOP protein expression enhances the viability of T cells from Gimap5^−/−^ rats. (A) Western blot analyses of the ER stress apoptotic factor CHOP in purified T cells transfected with either CHOP siRNA (siCHOP) or non-specific control siRNA (siControl). Shown are results of two separate knockdown experiments. Actin was used as a loading control. (B,C) Representative flow dot plots depicting cells positive for annexin V (vertical axis) and 7AAD staining (horizontal axis) on gated TCR^+^ or TCR^−^ lymphocytes. Bar graphs display the percentage of TCR^+^ or TCR^−^ lymphocytes that were positive for both annexin V and 7AAD staining (left), positive for annexin V staining alone (middle), and negative for both staining (right). Error bars represent the SEM of quadruplicate samples; data shown are representative of two independent experiments (*** *P*<.001).

CHOP protein plays an important role in ER stress-induced programmed cell death and was found to be increased only in *Gimap5^−/−^* BBDP rat T cells (Figures 2,4). To investigate a role for CHOP in apoptotic signaling of these T cells, we purified and transfected BBDP T cells with siRNAs targeted to CHOP (siCHOP) or non-specific control siRNA (siControl). Western blot analyses revealed a significant reduction in CHOP protein expression in T cells transfected with siCHOP as compared to siControl transfected T cells (Fig. 6A). The effects of reducing CHOP protein expression on induction of ER stress-induced apoptosis were examined by flow cytometry. Annexin V and 7AAD staining revealed three distinct populations of T cells: viable (annexinV^−^7AAD^−^, lower left quadrant), early apoptotic (annexinV^+^7AAD^−^, upper left quadrant), and late apoptotic and necrotic (annexinV^+^7AAD^+^, upper right quadrant) (Fig. 6B). As compared to T cells transfected with siControl, CHOP depletion significantly decreased the percentage of T cells in late apoptosis, with a concomitant increase in the percentage of non-apoptotic T cells (Fig. 6B). In contrast, the percentage of cells that were in the early stages of apoptosis were similar in T cells transfected with siCHOP or siControl (Fig. 6B).

To ensure that the results of CHOP depletion were specific to T cells, the effects of siCHOP transfection were also examined in TCR^−^ lymphocytes. In contrast to T cells, there were no statistical differences in the percentages of viable, early apoptotic, and late apoptotic TCR^−^ lymphocytes between siCHOP and siControl transfected cells (Fig. 6C). In their aggregate, these data suggest that CHOP protein actively participates in apoptotic signaling in *Gimap5^−/−^* T cells, and reduction of this ER stress apoptotic factor inhibits T cell death.

## Discussion

In this study we analyzed the ER stress response in T cells from *Gimap5^−/−^* BBDP rats. We demonstrate that absence of functional full length Gimap5 protein in T cells leads to ER stress response signaling as evidenced by increased GRP78 protein expression. In addition, our data show that BBDP T cells initiate ER stress-induced apoptotic signaling through upregulation of CHOP protein. Finally, decreasing the expression of this ER stress apoptotic factor, protected BBDP T cells from ER stress-induced death. These observations suggest that Gimap5 protein plays an essential role in the maintenance of ER homeostasis and is indispensable for T cell survival.

Lymphopenia in the BBDP rat was originally linked to a recessive mutation in a diabetes susceptibility locus designated *lyp/Iddm1*, which encodes for Gimap5 protein [9], [10]. Although spontaneous diabetes development in the BBDP rat depends on the presence of *lyp/Iddm1*, diabetes susceptibility and lymphopenia are traits that can be inherited independently [34]. To ensure that ER stress response signaling in T cells from the BBDP rat is due to absence of functional Gimap5 protein and not a byproduct of diabetes development, we only used nondiabetic BBDP rats for studies. Furthermore, flow cytometry analyses on *Gimap5^−/^* ^−^ T cells from congenic WF.*ART2a.Gimap5^−/−^* rats that do not develop spontaneous diabetes but carry the *Gimap5* null mutation have increased expression of ER stress response proteins, including GRP78 and CHOP (Figure S1E,F). Overall, this indicates that ER stress response signaling in T cells from the BBDP rat is a result of the absence of functional Gimap5 protein product and not a secondary consequence of spontaneous diabetes development.

Within the immune system, the establishment of immunological tolerance involves mechanisms to delete self-reactive T cells in order to avoid autoimmune diseases such as type 1 diabetes [35]. The interaction of TCRs on thymocytes with thymic stromal cells are important for proper T cell development and eventually allow for CD4^+^CD8^+^ double positive thymocytes to differentiate into mature CD4^+^CD8^−^ or CD4^−^CD8^+^ single positive thymocytes [36], [37]. Our data reveal that the percentage of double positive and single positive thymocytes does not differ between BBDR and BBDP rats, despite the absence of Gimap5 in the BBDP rat. Furthermore, ER stress response signaling is similar in the thymocyte populations from both BBDR and BBDP rats indicating the main ER regulatory functions for Gimap5 protein may occur during T cell post-thymic development. In general, our data indicate that ER stress response and ER stress-mediated signaling may only occur minimally during thymocyte development as levels of ER chaperones remained constant as double positive thymocytes matured to the single positive stage.

Following thymic development, T cells still expressing CD90, known as RTEs, enter the circulation. In the BBDP rat, a limited number of RTEs survive, down-regulate CD90 expression and become mature T cells, as others have reported and we have confirmed (Fig. 4A) [38]. Because RTEs undergo apoptosis rapidly in the *Gimap5^−/^* ^−^ BBDP rat, we initially hypothesized that ER stress protein levels would be higher in RTEs than in CD90^−^ mature T cells from the same animal. Our data suggest that ER chaperone expression is similar in all CD90^+^ and CD90^−^ *Gimap5^−/−^* T cell subpopulations in the BBDP rat. Our data further demonstrate that the percentage of the peripheral CD90^+^ and CD90^−^ T cells that express the activation marker CD25 is higher in the BBDP rat than in the BBDR rat (Fig. 5A,B). Although activation of T cells is known to enhance ER stress [32], [33], CD25^−^ T cell populations from the *Gimap5^−/−^* BBDP rat expressed ER stress levels significantly higher than their counterparts from the *Gimap5^+/+^* BBDR rat. These data indicate a global absence of the protein is sufficient to initiate ER stress response signaling throughout peripheral T cell development and that activation signals are not required for ER stress induction.

ER stress response signaling has been shown to be required for numerous physiological functions in several cell types [32], [33], [39], [40]. In addition, pathological signals from the ER have been attributed to cell death and apoptosis and linked to many diseases, including diabetes [18], [41]. In this report, we show that absence of functional Gimap5 protein leads to these pathological signals from the ER and subsequent ER stress-induced apoptosis in T cells from the BBDP rat. We suggest that triggering of the ER stress-induced apoptotic pathway in the T cells of BBDP rats is the underlying mechanism for their lymphopenia. This inference is supported by our data demonstrating that decreasing CHOP protein expression reduces the number of apoptotic T cells. Interestingly, although B cells normally express Gimap5, we found that ER stress response signaling in B cells is similar between *Gimap5^+/+^* BBDR and *Gimap5^−/−^* BBDP rats (Fig. 2B). These data suggest that modulation of ER stress response signaling by Gimap5 is required for the maintenance of T cell survival.

As documented above, this report demonstrates that ER stress-induced apoptosis occurs in *Gimap5^−/−^* T cells. However, we and others have previously shown a role for Gimap5 protein in the maintenance of mitochondrial integrity for T cell survival [11], [42]. These two Gimap5-dependent processes are both linked through CHOP, a protein that is both highly expressed in cells undergoing ER stress-induced cell death and also capable of sensitizing mitochondria to numerous apoptotic factors [43]. This sensitization occurs via downregulation of antiapoptotic Bcl-2 protein, activation of JNK and its associated proapoptotic downstream kinases [18], and activation of caspase-12 [44]. We hypothesize that CHOP protein through its role in ER stress-induced apoptotic signaling may lead to the disruption of mitochondrial integrity that is observed in *Gimap5^−/−^* T cells [11], [42]. We propose that loss of mitochondrial integrity in *Gimap5^−/−^* T cells may be secondary to ER dysfunction and subsequent ER stress-induced apoptotic signaling.

The intracellular distribution of Gimap5 protein remains controversial, but recent research indicates localization to the ER [12]. Our data support an intimate link between wild type Gimap5 and the ER due to ER stress response signaling in *Gimap5^−/−^* T cells. Accumulation of intracellular proteins is known to cause ER stress and can lead to cell death through CHOP signaling [45]. This association has been demonstrated in pancreatic beta cells of the Akita mouse in which accumulation of mutant insulin causes ER stress and eventual apoptosis through CHOP protein induction [46]. We observed an accumulation of αβTCR protein within *Gimap5^−/^* ^−^ BBDP rat lymphocytes that correlates with increased GRP78 levels (unpublished observations).

In the BBDP rat there is the frameshift mutation in *Gimap5* which results in a truncated protein product [12]. We can not rule out the possibility that the presence of this truncated protein product or increased intracellular αβTCR protein may accumulate in the ER and lead to the pathological ER stress that is observed in *Gimap5^−/−^* T cells. In either case, disruption of CHOP was effective in delaying apoptosis and preventing cell death, thus indicating the importance of CHOP in ER stress-associated T cell death signaling. Interestingly, overexpression of either wild type rat Gimap5 or the truncated BBDP rat Gimap5 in a rat T cell line is associated with enhanced apoptosis [42]. In this report, the apoptosis-inducing effect of mutant Gimap5 was much greater than that of the overexpressed wild type protein. In the context of our data, this may indicate that the expression of Gimap5 in T cells is both required for survival and tightly regulated.

In conclusion, our findings suggest a role for ER stress in mediating the spontaneous apoptosis found in T cells from *Gimap5^−/−^* rats. It will be of interest to determine if the same mechanisms are responsible for the T cell lymphopenia and hepatic dysfunction observed in *gimap5^−/−^* mice [5]. We provide the first evidence of a link between Gimap5 protein and the maintenance of ER homeostasis and cellular integrity. Our results indicate that proper functioning of the ER in T cells is critical for their survival. Investigations into the transient lymphopenia which occurs following virus infections in humans [47] and in the blood of patients with type 1 diabetes [48] may reveal a role for ER regulatory pathways in mediating human immunity. Further studies of wild type and mutant Gimap5 protein are needed to precisely define the mechanisms by which they affect T cell survival and function. Such studies may uncover novel pathways capable of modulating the survival and function of T cells and other tissues.

## Materials and Methods

### Animals

*Gimap5^−/−^* BBDP and *Gimap5^+/+^* BBDR rats were obtained from Biomedical Research Models (Worcester, MA). BBDP rats are T cell lymphopenic in both the CD4 and to an even greater extent in the CD8 compartments; BBDR rats have a normal peripheral lymphocyte phenotype [30]. Eight week-old male or female rats were used, and all rats were nondiabetic at the time of study. Congenic WF.*ART2a.Gimap5^−/−^* rats were also developed by us by crossing WF (Charles River Breeding Laboratories, Wilmington, MA) and BBDP rats. The intercross was followed by 8 backcrosses to WF before intercrossing to establish homozygosity for both the *Iddm14* [49] and *lyp* (*Gimap5*) loci on chromosome 4 and the *ART2a* locus [50] on chromosome 1. The WF.*ART2a.Gimap5^−/−^* rat bears no BB markers except in the regions of chromosome 4 bounded by *D4Rat101-D4Rat58* and chromosome 1 bounded by *D1Rat29* and *D1Rat287*, and it does not develop spontaneous diabetes (unpublished observations). Control WF.*ART2a* congenic rats were developed by us and differ from ordinary non-lymphopenic, non-diabetic WF animals in that they express the “a” rather than the “b” allotype of the ART2 T cell alloantigen on chromosome 1 [50], [51]. WF.*ART2a* are nondiabetic and homozygous for wild type *Gimap5*. All animals were housed in a viral antibody-free facility and maintained in accordance with the guidelines of the University of Massachusetts Medical School Institutional Animal Care and Use Committee and the *Guide for the Care and Use of Laboratory Animals* (Institute of Laboratory Animal Resources, National Research Council, National Academy of Sciences, 1996).

### Thymi and lymph node cell preparation

Thymi and cervical and mesenteric lymph nodes were removed from BBDR and BBDP rats, processed aseptically, and extruded through mesh sieves. Single cell suspensions for flow cytometry were prepared as described below.

### Western blotting

Thymi, lymphocytes, or purified T cells were lysed [52] and protein concentrations determined by bicinchoninic acid (BCA) protein assay (Sigma-Aldrich, St. Louis, MO). Protein (30 µg) was mixed with 4× SDS-PAGE loading buffer and Western blot analyses were performed as described [52]. Actin was used as a loading control.

### Antibodies

Anti-rat CD8a-PE (clone Ox-8), FITC or PerCp-conjugated anti-rat TCRαβ (clone R73), anti-rat CD90-FITC (clone HIS51), Biotin-conjugated anti-rat CD25 (clone OX-39), and Streptavidin-conjugated APC-Cy7 mAbs were obtained from BD Biosciences (San Diego, CA). Purified GRP78 mAbs (clone 40) and isotype control Abs were also obtained from BD Biosciences. A Zenon Mouse IgG2a or IgG1 Labeling Kit, Alexa Fluor 647 (Invitrogen, Carlsbad, CA), was used to label purified GRP78 or CHOP mAbs per the manufacturer's directions. Anti-rat CD4-Pacific Blue mAb and its corresponding isotype control Ab were obtained from Serotec (Raleigh, NC). Anti-actin (clone C4) antibody was obtained from Chemicon International (Temecula, CA). Anti-ERp72 and anti-GRP94 were obtained from Calbiochem (San Diego, CA) and Stressgen (San Diego, CA), respectively. Cleaved capase-3 antibody was obtained from Cell Signaling Technology (Danvers, MA). Polyclonal antiserum to Gimap5 was generated by us as described [11]. Anti-CHOP antibody and anti-mouse IgG horseradish peroxidase (HRP) conjugate were obtained from Santa Cruz Biotechnology (Santa Cruz, CA).

### Flow cytometry

Single cell suspensions from cervical and mesenteric lymph node cells were washed and suspended in PBS containing 1% fetal clone serum (HyClone, Logan, UT) and 0.1% sodium azide (Sigma-Aldrich). Samples were washed, incubated for 20 min with fluorescent mAbs to various cell-surface markers as described in Results. To detect intracellular GRP78 or CHOP protein, cells were permeabilized using Cytofix/Cytoperm (BD Biosciences) according to the manufacturer's directions. Cells were washed and incubated with Alexa Fluor 647-conjugated GRP78 or CHOP mAb for 20 min. Labeled cells were washed, fixed with 1% paraformaldehyde (Polysciences, Warrington, PA) in PBS and analyzed using an LSR II instrument (BD Biosciences, San Jose, CA) and FlowJo Software (PC version 7.2.2; Tree Star, Ashland, OR). Lymphoid cells were gated according to their light-scattering properties.

### T cell transfection

T cells from BBDP rats were purified as described [52]. T cells were plated at 6×10^6^ cells per well in 3 mL of RPMI medium (Sigma-Aldrich) containing 10% FBS (Hyclone), 1% Pen/Strep/Glut (Gibco, Carlsbad, CA), and 0.1% β-mercaptoethanol (Gibco) at 37°C in the presence of 10 ng/mL PMA (Calbiochem) and 100 ng/mL ionomycin (Calbiochem). After 12 h, cells were resuspended in 100 µL of Nucleofector solution using human T cell Nucleofector kit (VPA-1002, Amaxa Biosystems, Gaithersburg, MD) with 100 nM rat CHOP siRNA (catalogue no. L-088282-01, Dharmacon, Lafayette, CO) or 100 nM control siRNA (catalogue no. D-001210-02, Dharmacon) and nucleofected using program U-014 in the Amaxa Nucleofector apparatus. Following nucleofection, T cells were plated in 3 mL complete media and harvested at 30 h for protein preparation and flow cytometry analysis.

### Apoptosis Detection

To measure cellular apoptotic signaling, transfected T cells were harvested, washed twice with PBS, and reacted with PE-conjugated anti-annexin V and 7-amino-actinomycin D (7AAD) according to the manufacturer's directions (BD Pharmingen). At least 20,000 cells per sample were analyzed using a FACSCalibur (BD Biosciences) and FlowJo software.

### Statistics

Statistical analyses were performed with GraphPad Prism software (Graphpad Software, San Diego, CA). Parametric data are presented as the mean±the standard error of measurement (SEM) and were analyzed using two-tailed unpaired *t*-tests. Values of *p*<0.05 were considered statistically significant.

## Supporting Information

Figure S1 T cell populations from congenic WF.*ART2a.Gimap5^−/−^* rats that do not develop spontaneous diabetes exhibit enhanced ER stress response signaling. Representative analyses indicating the gating of TCR^+^ (A) and CD4^+^ or CD8^+^ (B) lymphocytes within congenic WF.*ART2a.Gimap5^−/−^* (Type 31) and control congenic WF.*ART2a.Gimap^+/+^* (Type 20) rats. Representative flow dot plots depicting CD90 expression (horizontal axis) and forward scattering (vertical axis) of lymphocytes within gated CD4^+^ and CD8^+^ T cell populations from Type 31 (C) or Type 20 (D) rats. Numbers represent the percentage of cells (±SEM of triplicate samples) in each gate shown. Intracellular GRP78 (E) and CHOP (F) expression in RTEs (CD90^+^) and mature T cells (CD90^−^) from Type 31 (black line) and Type 20 rats (shaded region). Depicted in each histogram is the isotype control (dotted line). The MFI of GRP78 or CHOP protein expression is displayed in bar graphs with error bars representing the SEM of duplicate samples. Data shown are representative of two independent experiments (*P<.05; **P<.01; ***P<.001).
(0.94 MB TIF)
