# Supplementary figures and images for: CHOP Mediates Endoplasmic Reticulum Stress-Induced Apoptosis in Gimap5-Deficient T Cells
Source: PLoS One. 2009 May 8;4(5):e5468. doi: 10.1371/journal.pone.0005468 (PMC2674944; doi:10.1371/journal.pone.0005468)

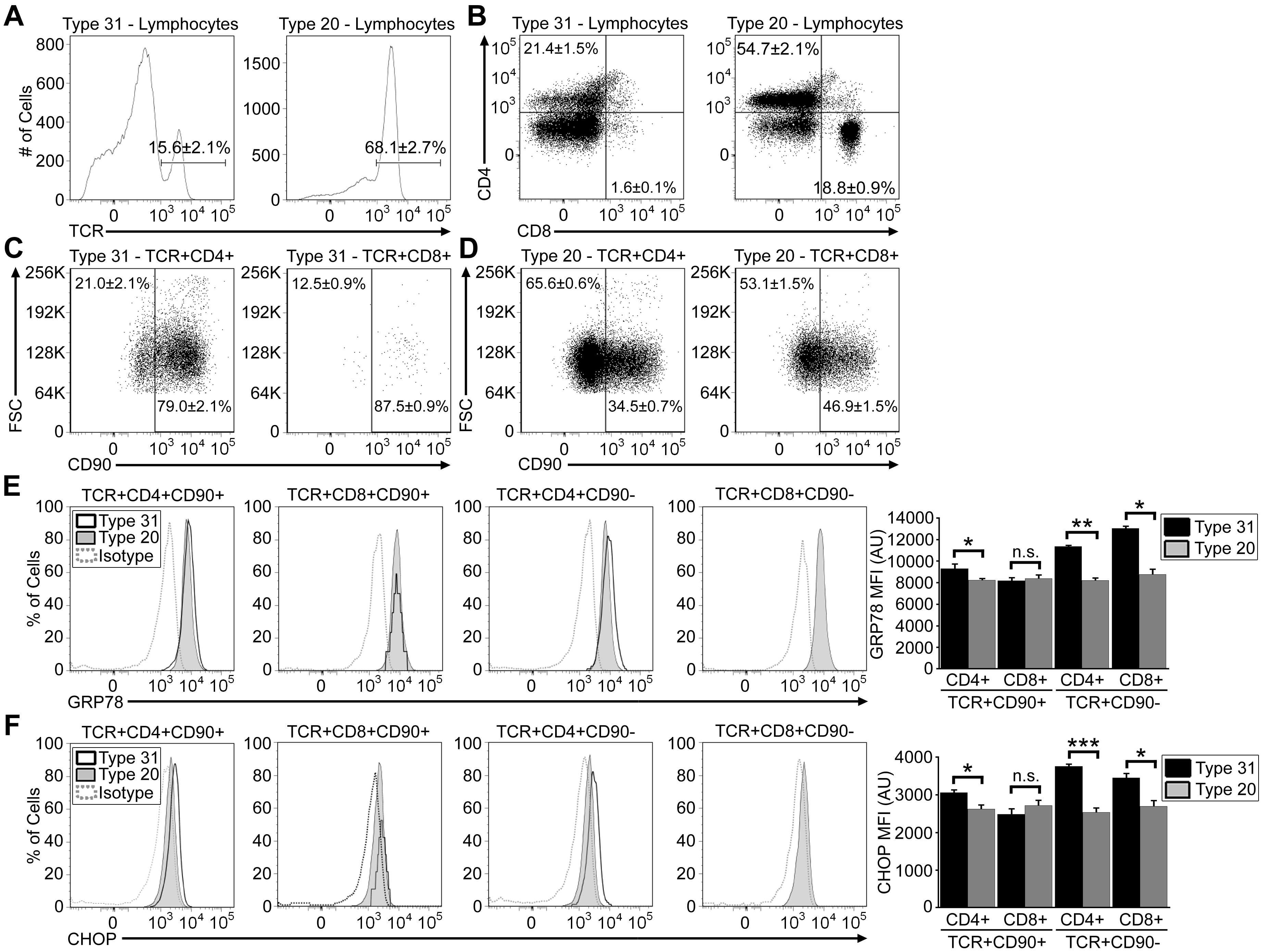

Supplement: Figure S1 — T cell populations from congenic WF.ART2a.Gimap5−/− rats that do not develop spontaneous diabetes exhibit enhanced ER stress response signaling. Representative analyses indicating the gating of TCR+ (A) and CD4+ or CD8+ (B) lymphocytes within congenic WF.ART2a.Gimap5−/− (Type 31) and control congenic WF.ART2a.Gimap+/+ (Type 20) rats. Representative flow dot plots depicting CD90 expression (horizontal axis) and forward scattering (vertical axis) of lymphocytes within gated CD4+ and CD8+ T cell populations from Type 31 (C) or Type 20 (D) rats. Numbers represent the percentage of cells (±SEM of triplicate samples) in each gate shown. Intracellular GRP78 (E) and CHOP (F) expression in RTEs (CD90+) and mature T cells (CD90−) from Type 31 (black line) and Type 20 rats (shaded region). Depicted in each histogram is the isotype control (dotted line). The MFI of GRP78 or CHOP protein expression is displayed in bar graphs with error bars representing the SEM of duplicate samples. Data shown are representative of two independent experiments (*P<.05; **P<.01; ***P<.001). (0.94 MB TIF) [file pone.0005468.s001.tif]
